# Successful dental implant rehabilitation in a posterior mandibular site affected by florid cemento-osseous dysplasia: A case report

**DOI:** 10.34172/japid.026.4070

**Published:** 2026-02-22

**Authors:** Nima Naddafpour, Hassan Semyari, Hadise Semyari, Rozhan Khaledtaj, Hossein Semyari

**Affiliations:** ^1^Department of Periodontics, TeMS.C., Islamic Azad University, Tehran, Iran; ^2^Department of Periodontics, Faculty of Dentistry, Shahed University, Tehran, Iran; ^3^Heuma Training Center, Tehran, Iran

**Keywords:** FCOD, Florid cemento-osseous dysplasia, Implant

## Abstract

Florid cemento-osseous dysplasia (FCOD) is a benign fibro-osseous disorder characterized by altered and poorly vascularized bone, which complicates implant placement by increasing the risk of infection and impaired healing. This case report documents the successful placement and one-year follow-up of two dental implants in the posterior mandible of a 45-year-old female patient with FCOD. Radiographic and clinical evaluations confirmed stable osseointegration and successful functional restoration, with no complications or changes in lesion size during follow-up. These findings suggest that, with careful patient selection, the use of atraumatic surgical techniques, meticulous infection control, appropriate postoperative oral care, and consistent follow-up, dental implant therapy can be successfully performed in selected cases of FCOD. However, due to the limited reported cases, extensive longitudinal studies are required to guide implant therapy in patients with FCOD.

## Graphical Abstract

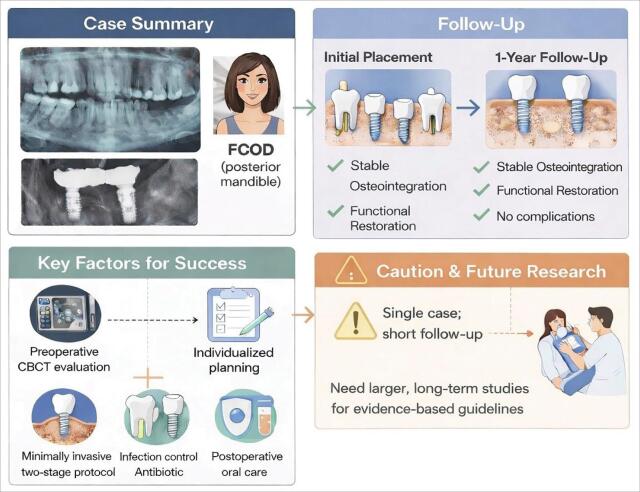

## Introduction

 The most effective method of oral rehabilitation is dental implants; however, the quality of the surrounding bone is a key factor in treatment success. Dental implants are considered a controversial treatment option for those with dysplastic bone diseases such as fibro-osseous dysplasia. Bone structural alterations that affect its normal blood supply and plasticity are frequently linked to the inherent character and biological behavior of bone dysplasia, which may further complicate osseointegration.^1^

 Cemento-osseous dysplasia (COD) comprises a group of fibro-osseous lesions of the jaws with diverse clinical subtypes, which can occur at various anatomical sites and in varying sizes.

 COD occurs more frequently in middle-aged African American women, with a higher tendency to involve the mandible. A hallmark of COD is the replacement of normal bone with fibrous or cementum-like tissue.^2^ Given the diagnostic importance of X-ray imaging in FCOD, performing a biopsy or histopathological examination is generally unnecessary. It should be noted that a biopsy may increase the risk of infection or jaw fracture, potentially leading to adverse effects on the patient’s overall health.^3^

 Based on the histopathologic spectrum and the extent of jaw involvement, osseous dysplasia has been further categorized into florid, focal, and periapical dysplasia. Florid cemento-osseous dysplasia (FCOD) is a rare benign fibro-osseous lesion primarily in the posterior mandible. The pathogenesis of the disease involves the replacement of normal bone with fibroblasts, collagen fibers, and immature bone tissue.^4,5^ Although generally asymptomatic, infected FCOD is associated with the development of bone sequestrum and symptoms such as pain and purulent secretions. Exposure of the lesion in the oral cavity, focal expansion, and facial deformity may occur, leading to subsequent necrosis.^6^ This altered bone structure results in hypovascularity and reduced remodeling capacity, posing challenges for dental implant placement.

 Several reports have described implant therapy in patients with FCOD, with varied outcomes, ranging from successful osseointegration to early failure due to infection or poor vascularization of the affected bone. This inconsistency highlights a persistent knowledge gap, as evidence on the long-term success and stability of implants in FCOD-affected bone remains limited.

 This study reports the successful osseointegration of an implant in a florid cemento-osseous lesion area with one-year follow-up.

## Case Report

###  Patient Presentation, Diagnosis, and Treatment Planning

 A 45-year-old Caucasian woman visited the Periodontology Department of the Islamic Azad University Dental School in Tehran, with a chief complaint of unilateral posterior mandibular edentulism, seeking evaluation for implant placement in the affected area. The patient’s medical history revealed no systemic diseases, ongoing medications, or significant family medical history.

**Figure 1:**
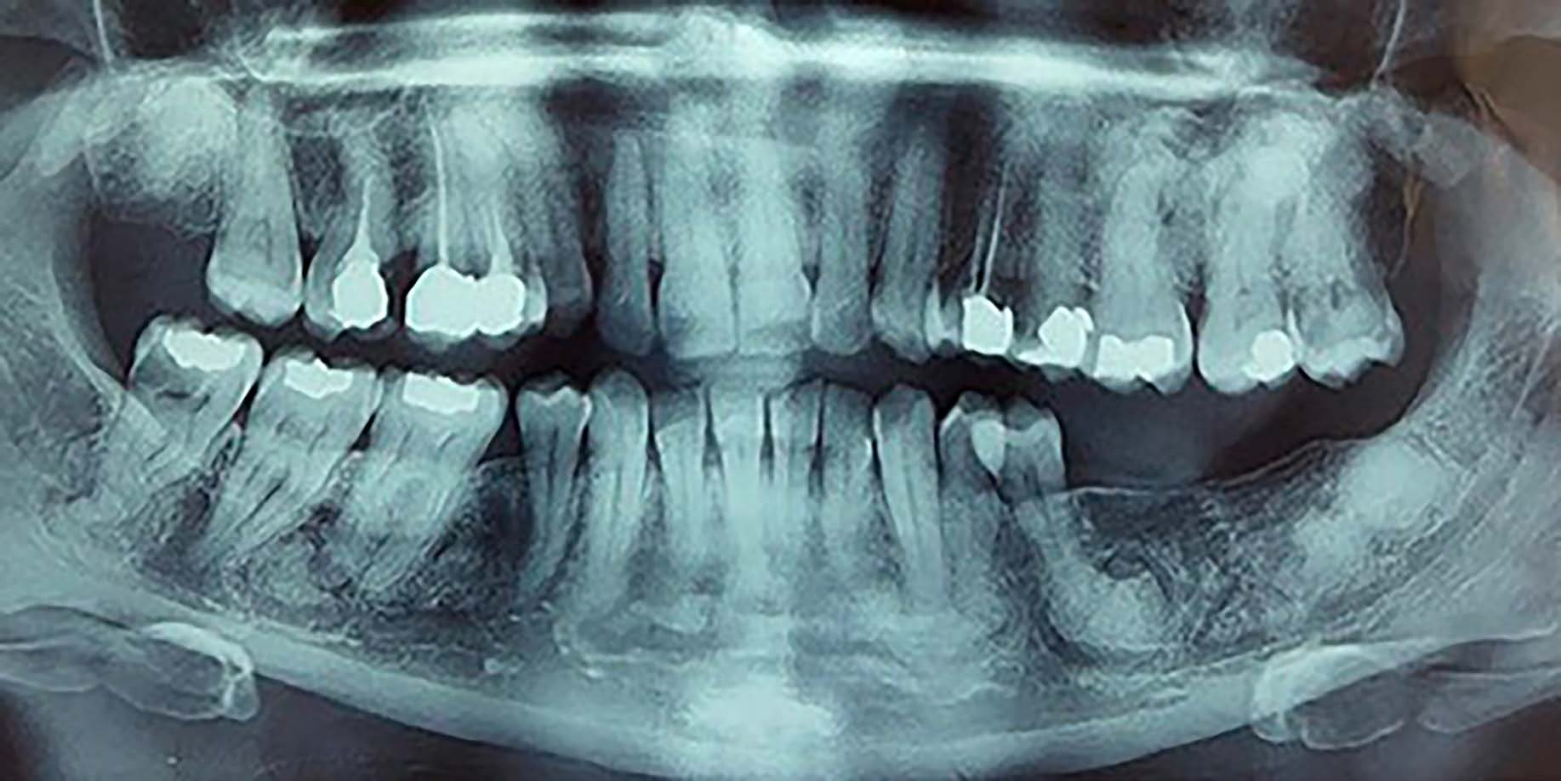
Panoramic radiograph showing FCOD lesions in the maxilla and mandible

**Figure 2:**
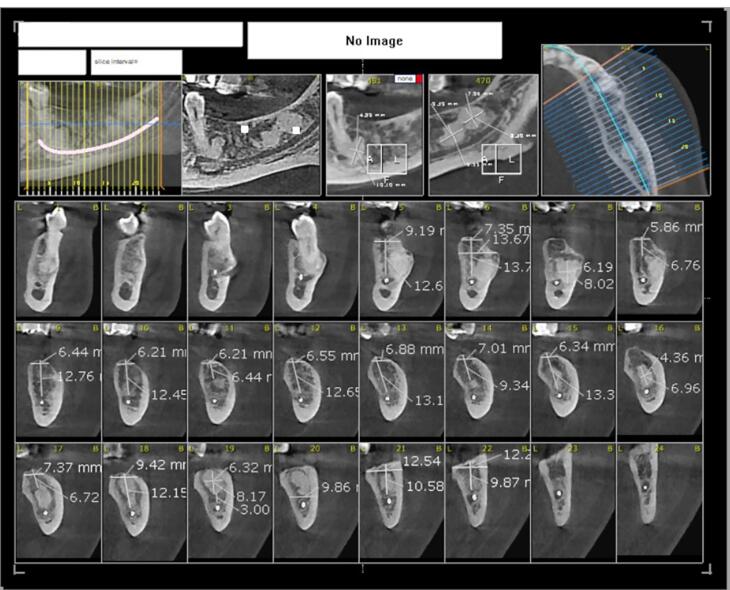
Pre-oprative CBCT view illustrating the extent and location of FCOD lesions

 Panoramic radiography revealed multiple opaque lesions, some with radiolucent margins, in the periapical regions of the maxilla and mandible, while the teeth remained vital (Figure 1). CBCT scans showed no evidence of root resorption (Figure 2). Radiographic findings were suggestive of florid cemento-osseous dysplasia (FCOD). As FCOD diagnosis is typically based on radiographic and clinical findings, the patient was referred to a radiologist for confirmation, and the diagnosis was verified.

 Potential complications were explained to the patient, and after obtaining written informed consent, an implant treatment plan was developed in consultation with the Prosthodontics Department. Written informed consent was obtained from the patient for publication of this case and accompanying images, and the study was conducted in accordance with the Declaration of Helsinki.

###  Surgical Procedures and Clinical Management

 Stringent infection control measures were implemented to minimize the risk of secondary infection. One hour before surgery, the patient received 2 g of oral co-amoxiclav for prophylaxis to reduce the risk of surgical site infection, followed by 500 mg every 8 hours for one week after surgery. The patient rinsed with chlorhexidine mouthwash both before and after surgery and continued using it twice daily for 2 weeks. Surgical site infection control was ensured, and the procedure was performed in the shortest possible time.

 A two-stage surgical placement of two implants was performed in the regions of the left mandibular second premolar and first molar. Local anesthesia was achieved using 2% lidocaine with inferior alveolar and long buccal nerve block techniques, avoiding local infiltration to minimize the risk of ischemia at the surgical site. An intrasulcular incision was made around the first premolar with distal crestal extension. Full-thickness buccal and lingual flaps were elevated. The lingual flap was minimally reflected, maintaining its periosteal continuity to support healing. Osteotomy was performed according to the manufacturer’s standard drilling protocol (implantswiss, Switzerland) using sharp drills, up to the final drill size corresponding to the implant diameter (4.3*6 mm and 4.3*10 mm). Continuous external irrigation with normal saline was used during osteotomy to minimize thermal damage.

 Before implant placement, a brief pause ensured intra-osteotomy bleeding. The implants were placed with an appropriate insertion torque of approximately 35 N/cm^2^, and the cover screws were securely tightened. The flaps were sutured with 4-0 nylon. Although the insertion torque allowed for potential single-stage placement, a two-stage approach was chosen to enhance blood supply, improve healing, avoid premature loading, and reduce infection risk. Immediate postoperative periapical radiography was performed.

###  Postoperative Care and Follow-up

 After surgery, a detailed care plan for the patient, emphasizing the importance of good oral hygiene and dietary recommendations, was developed. 0.12% chlorhexidine gluconate mouthwash was prescribed. For managing pain effectively, a regimen of alternating doses of 400 mg ibuprofen and 500 mg acetaminophen, in addition to the necessary antibiotic, was prescribed. The patient was advised to rinse with 15 mL of mouthwash twice a day for 30 seconds. The antibiotic regimen consisted of co-amoxiclav (500 mg) every 8 hours for one week. The patient was instructed to avoid placing any stress on the surgical site to promote optimal healing. We arranged regular follow-up visits to monitor recovery and ensure the patient’s satisfaction.

**Figure 3:**
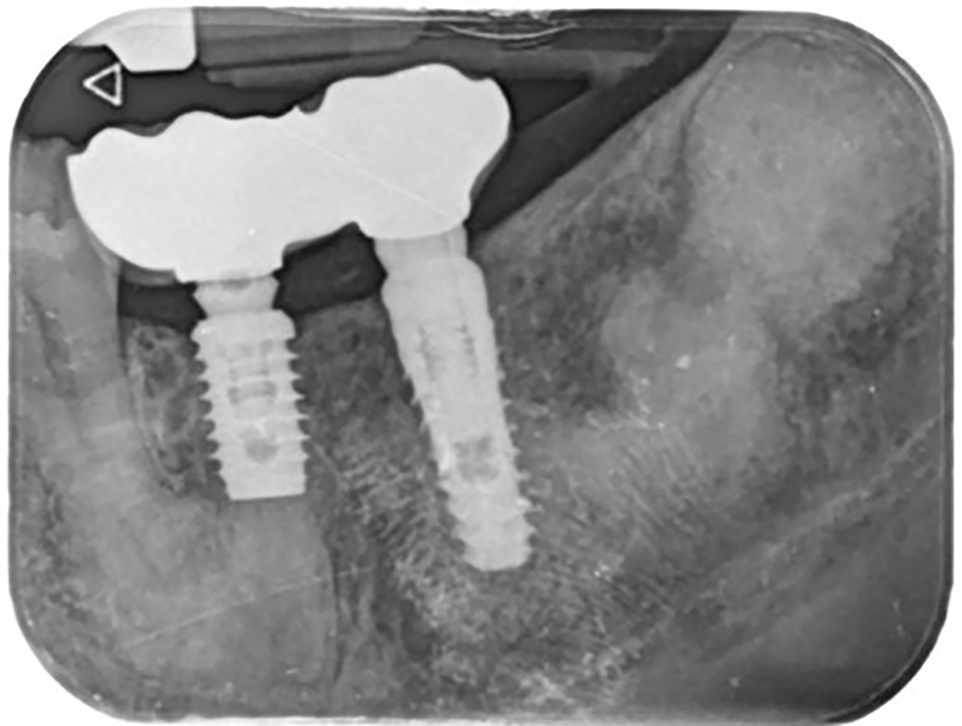
Periapical radiograph at one-year follow-up showing successful implant placement, stable osseointegration, and functional restoration

 A 3-month follow-up with radiographic evaluation confirmed proper implant integration and no changes in the size or characteristics of the FCOD lesions. The implants were then loaded with fixed prostheses. Additional follow-up radiographs at 6- and 12-month postoperative intervals confirmed implant integration and successful rehabilitation of the edentulous area, with no changes in the FCOD lesions (Figure 3). Clinical examination revealed no signs of complications, including soft-tissue inflammation, fistulas, probing depths exceeding 4 mm, or mobility. Bone levels remained optimal, the implants were functional, and both the patient and clinician were satisfied with the treatment outcomes.

## Discussion

 Elective surgical interventions, including tooth extractions, are generally discouraged in patients with FCOD due to the risk of poor healing, sequestrum formation, infection, mandibular fracture, and osteomyelitis.^7-9^ Waldron et al.^10^ reported compromised healing and sequestrum formation following extractions near COD lesions. Implant failure in FCOD patients is primarily attributed to localized necrosis due to thermal damage during drilling and compromised vascularity, which limits bone regeneration and increases infection risk.^11^ Additionally, due to the avascular nature of FCOD lesions, antibiotic therapy is often ineffective in managing postoperative infections.^12^ However, it appears that strict infection control and appropriate antimicrobial prophylaxis contributed significantly to the successful implant treatment in this case.

 Tsai et al^13^ and Sukegawa et al^14^ have suggested surgical removal of FCOD lesions before implant placement to mitigate associated risks. However, in the present case, due to the extent of the lesions, the potential risk of jaw weakening or fracture, and the patient’s preference, surgical removal was not pursued.

 Gerlach et al^15^ reported implant failure in a patient with FCOD and cemento-ossifying fibroma, attributing the failure to lesion perforation during implant placement, a view supported by Esfahanizadeh and Yousefi.^5^ Nonetheless, studies examining implants placed directly in FCOD-affected bone demonstrate that osseointegration is possible under careful surgical planning. Jagtap et al^4^ reported stable implants in a mandibular FCOD lesion over a five-year follow-up, emphasizing meticulous surgical technique as critical for success. Similarly, Oliveira et al^16^ documented a stable implant in an FCOD lesion over three years, highlighting the importance of vascular assessment to reduce infection risk.^1,16^ Shadid and Kujan^17^ noted minor inflammatory complications in one of three implants placed within lesions, though all achieved osseointegration.^17^ Park et al^18^ reported a case of long-term implant survival within an FCOD lesion, which eventually failed after 16 years due to peri-implantitis. Histologic and tomographic evaluations confirmed successful integration, with cementum-like tissue (CLT) directly contacting the implant surface, suggesting a unique form of osseointegration.^18^ These findings suggest that while implants in FCOD lesions can succeed, the risk of delayed healing and postoperative complications remains higher compared to normal bone.

 However, implants placed in adjacent unaffected bone consistently demonstrate favorable outcomes.^16^ Li et al^19^ reported high survival rates with minimal complications in implants positioned in normal bone adjacent to FCOD lesions. Even in mixed cases where implants were placed both in lesion and normal sites, implants in unaffected bone remained stable, whereas those in lesions sometimes experienced delayed healing.^20^ The preservation of vascular supply and normal bone quality likely accounts for the improved predictability and long-term success of these implants.

 In a systematic review, Hosseinpour et al^1^ concluded that implant therapy, when approached conservatively and with thorough planning, may be a feasible option for patients with FCOD. However, the limited number of cases prevents definitive conclusions.

 The comparative success rates are summarized in Table 1, which underscores the clinical distinction between implants in dysplastic versus normal bone. While isolated successful cases exist within FCOD lesions, overall complication rates, including infection, implant mobility, and delayed osseointegration, are significantly higher than those in adjacent normal bone. Advanced imaging, such as cone-beam computed tomography, allows clinicians to precisely evaluate lesion extent and select the most suitable implant sites, enhancing predictability.^21^

**Table 1:** Reported clinical outcomes of dental implants in patients with cemento-osseous dysplasia

**Study**	**Patients / Implants**	**Implant Site**	**Follow-up**	**Complications / Outcomes**
Japtab et al.^4^	multiple patients, 17 implants	Mandible, FCOD lesion	up to 5 years	Implants stable, no infection; meticulous technique crucial, Mobility in one implant after 26 months
Li et al.^19^	multiple patients, 11 implants	Adjacent normal bone, FCOD lesion	≥ 3 years	Overall survival 91% Implants outside lesions perform better
Oliveira et al.^16^	1 patient, 1 implant	FCOD lesion	3 years	Implant stable; highlighted infection risk if vascularity compromised
Hosseinpour et al.^1^	3 patients, 5 implants	FCOD lesion	up to16 years	One failed after 16 years due to pre-implantitis
Graf et al.^20^	1 patient, 3 implants	Adjacent normal bone, FCOD lesion	13 months	The implant in the lesion had delayed healing; normal bone implants stable
Hu et al.^21^	multiple patients, 29 implants	COD affected regions	median 25 months	Overall success rate 79.3% All failures occurred in FLCOD Focal COD achieved 100% success Stage III lesions showed higher success

## Conclusion

 Although dental implants can be successfully placed in bone affected by FCOD, placement in unaffected adjacent bone remains the preferred option. Precise preoperative evaluation, individualized treatment planning, minimally invasive implant-site technique, two-stage implant protocol, meticulous infection control, prophylactic antibiotic coverage, and comprehensive postoperative oral care are key factors contributing to the successful management of this case. However, the favorable outcome observed should be interpreted with caution, given the inherent limitations of a single case report and the relatively short follow-up period. Well-designed prospective studies, including larger longitudinal studies, are needed to establish evidence-based guidelines for implant placement in patients with FCOD.

## Competing Interests

 The authors declare that they have no competing interests regarding the authorship and/or publications of this paper.

## Consent to Publication

 Written inform consent was obtained from the patient for the publication of this case report and any accompanying images.

## Data Availability

 All data supporting the findings of this case report are included within the article.

## Ethical Approval

 Not applicable.

## References

[1] Hosseinpour S, Khademi MH, Erfani M, Mosaddad SA, Heboyan A (2024). Are implant-based treatments considered viable for patients with focal or florid cemento-osseous dysplasia? A systematic review. Maxillofac Plast Reconstr Surg.

[2] Decolibus K, Shahrabi-Farahani S, Brar A, Rasner SD, Aguirre SE, Owosho AA (2023). Cemento-Osseous Dysplasia of the Jaw: Demographic and Clinical Analysis of 191 New Cases. Dent J (Basel).

[3] Das BK, Das SN, Gupta A, Nayak S (2013). Florid cemento-osseous dysplasia. J Oral Maxillofac Pathol.

[4] Jagtap R, Gupta S, Bhat M, Mehta N, Gupta S (2024). Dilemma with implant placement in patients with florid cemento-osseous dysplasia: A literature review. Sci Prog.

[5] Esfahanizadeh N, Yousefi H (2018). Successful Implant Placement in a Case of Florid Cemento-Osseous Dysplasia: A Case Report and Literature Review. J Oral Implantol.

[6] Bastos YVP, Carlos R, Oliveira PT, Pires BC, Cangussu MCT, Xavier FCA (2022). Florid cemento-osseous dysplasia-related osteonecrosis: A series of cases. Ann Diagn Pathol.

[7] Sarmento DJ, Monteiro BV, de Medeiros AM, da Silveira EJ (2013). Severe florid cemento-osseous dysplasia: a case report treated conservatively and literature review. Oral Maxillofac Surg.

[8] Gündüz K, Avsever H, Karaçayli U, Senel B, Pişkin B (2009). Florid cemento-osseous dysplasia: a case report. Braz Dent J.

[9] Groot RH, van Merkesteyn JP, Bras J (1996). Diffuse sclerosing osteomyelitis and florid osseous dysplasia. Oral Surg Oral Med Oral Pathol Oral Radiol Endod.

[10] Waldron CA, Giansanti JS, Browand BC (1975). Sclerotic cemental masses of the jaws (so-called chronic sclerosing osteomyelitis, sclerosing osteitis, multiple enostosis, and gigantiform cementoma. Oral Surg Oral Med Oral Pathol.

[11] Shnaiderman-Shapiro A, Dayan D, Buchner A, Schwartz I, Yahalom R, Vered M (2015). Histopathological spectrum of bone lesions associated with dental implant failure: osteomyelitis and beyond. Head Neck Pathol.

[12] Bencharit S, Schardt-Sacco D, Zuniga JR, Minsley GE (2003). Surgical and prosthodontic rehabilitation for a patient with aggressive florid cemento-osseous dysplasia: a clinical report. J Prosthet Dent.

[13] Tsai WC, Wu YC, Ohiro Y, Fang CY (2024). Dental implants could be considered as a reliable treatment in florid cemento-osseous dysplasia patients after total excision. J Dent Sci.

[14] Sukegawa S, Kanno T, Kawai H, Shibata A, Matsumoto K, Sukegawa-Takahashi Y (2016). Surgical treatment and dental implant rehabilitation after the resection of an osseous dysplasia. J Hard Tissue Biol.

[15] Gerlach RC, Dixon DR, Goksel T, Castle JT, Henry WA (2013). Case presentation of florid cemento-osseous dysplasia with concomitant cemento-ossifying fibroma discovered during implant explantation. Oral Surg Oral Med Oral Pathol Oral Radiol.

[16] Oliveira MTF, Cardoso SV, Silva CJ, Zanetta-Barbosa D, Loyola AM (2014). Failure of dental implants in cemento-osseous dysplasia: a critical analysis of a case. Rev Odontol UNESP.

[17] Shadid R, Kujan O (2020). Success of dental implant osseointegration in a florid cemento-osseous dysplasia: A case report with 8-year follow-up. Clin Pract.

[18] Park WB, Han JY, Jang JS, Kang KL, Kang P (2019). Long-Term Implant Survivability of an Implant Having Direct Contact with Cementum-Like Tissue in a Preexisting Mandibular Intraosseous Lesion with a 16-Year Longitudinal Follow-up. Int J Periodontics Restorative Dent.

[19] Li S, Delgado-Ruiz R, Romanos G (2024). Dental implants versus removable prostheses for the management of edentulous sites in patients with florid cemento-osseous dysplasia: A systematic review of literature with a follow-up period of at least 3 years. Int J Oral Implantol (Berl).

[20] Graf T, Grogniet F, Contrepois M, Fricain JC, Catros S, Fenelon M (2025). Bone Regeneration and Dental Implant Surgeries in Florid Cemento-Osseous Dysplasia: A Case Report. J Oral Implantol.

[21] Hu H, Liu L, Man Y, Ye Z, You M (2025). Clinical and radiologic outcomes of dental implants in cemento-osseous dysplasia: a systematic review and retrospective case series. BMC Oral Health.

